# Fecal Protein Profile in Eight Dogs Suffering from Acute Uncomplicated Diarrhea before and after Treatment

**DOI:** 10.3390/vetsci10030233

**Published:** 2023-03-20

**Authors:** Matteo Cerquetella, Sara Mangiaterra, Giacomo Rossi, Alessandra Gavazza, Andrea Marchegiani, Gianni Sagratini, Massimo Ricciutelli, Simone Angeloni, Licia Fioretti, Carlotta Marini, Stefania Pucciarelli, Silvia Vincenzetti

**Affiliations:** 1 School of Biosciences and Veterinary Medicine, University of Camerino, Via Circonvallazione 93/95, 62024 Matelica, Italy; 2 Independent Researcher, 60121 Ancona, Italy; 3 School of Pharmacy, University of Camerino, Via Sant’Agostino 1, 62032 Camerino, Italy; 4 Chemistry Interdisciplinary Project (ChIP) Laboratory of LC-MS, University of Camerino, 62032 Camerino, Italy; 5 Independent Researcher, 60121 Ancona, Italy; 6 School of Biosciences and Veterinary Medicine, University of Camerino, Via Gentile II da Varano, 62032 Camerino, Italy

**Keywords:** dog, acute diarrhea, biomarker, fecal proteomics, patient monitoring

## Abstract

**Simple Summary:**

Little is known about what happens in the canine gastrointestinal tract during episodes of acute diarrhea in dogs. In the present study, a proteomic approach was used to investigate at the time of inclusion, and at two subsequent time points (2 and 14 days after the onset of the clinical condition), changes in the digestive environment in a subset of dogs suffering from acute uncomplicated diarrhea. Four (groups of) proteins showed significant differences at two or more of the three time points investigated, mainly evidencing a reaction of the organism to the disease. Further studies are needed to confirm the present findings.

**Abstract:**

Acute diarrhea is a very frequent condition affecting dogs; nevertheless, little is known about what happens in the GI tract during such conditions. Proteomics allows the study of proteins present in a specific biologic substrate, and fecal proteomic investigations have been recently implemented to study GI diseases in dogs. In the present study, the fecal protein profiles of eight dogs suffering from acute uncomplicated diarrhea at the time of inclusion was investigated for the first time, and then the same patients were followed, replicating two further evaluations at two subsequent time points (after 2 and 14 days from the first presentation), with the aim of gaining possible new insights regarding the pathologic changes in the gastrointestinal environment during such conditions. Two-dimensional gel electrophoresis (2-DE) was performed, followed by mass spectrometry. Nine spots, corresponding to four (groups of) proteins (i.e., albumin, alkaline phosphatase, chymotrypsin-C-like, and some immunoglobulins), showed significant differences at two or more of the three time points investigated, almost all behaving similarly and decreasing at T1 (2 days after the onset of the condition) and significantly increasing at T2 (14 days after the onset), mainly evidencing a reaction of the organism. Further studies including a greater number of patients and possibly different techniques are needed to confirm the present findings.

## 1. Introduction

Acute diarrhea is one of the most frequently reported daily complaints in a small animal veterinary clinic. It only rarely reveals a specific etiology, and the clinical presentation is extremely variable, ranging from uncomplicated cases (acute uncomplicated diarrhea—AUD) to life-threatening ones [1]; consequently, the therapy is also extremely variable and mostly based on symptomatic/supportive (seldomly etiologic) therapy, depending on the severity of the clinical signs and on the presence/absence of comorbidities [2,3,4]. Little is known about what happens in the gastrointestinal lumen/mucosa during acute diarrhea in living subjects [5,6,7].

Proteomics is the study of proteins present in a specific biological substrate. It has been recently applied in the field of veterinary gastroenterology through the study of the fecal proteome in healthy dogs and cats, and in dogs suffering from chronic enteropathies, as well as in cheetahs [8,9,10,11,12]. In one study in cats, the gastrointestinal (GI) mucosal proteome was also characterized in patients with inflammatory bowel disease, alimentary small cell lymphoma, and healthy controls [13]. Among the focal purposes of proteomics, there is the discovery of biomarkers to be applied for diagnostic/monitoring purposes, and more in general for a better understanding of the pathophysiology of determined conditions in specific biological environments. There is a growing interest in the search for new biomarkers useful in the diagnosis, course evaluation, and prognosis of patients with acute diarrhea [1,5,14,15].

The main aim of the present study was to evaluate, for the first time (to the authors’ knowledge), the fecal proteome of dogs suffering from AUD in order to gain possible new insights regarding the pathologic changes in the gastrointestinal environment during such conditions. The second aim was to evaluate, for the first time again, the evolution of the fecal protein profile during treatment and after healing, precisely after 2 (T1) and 14 days (T2). The choice to use feces as a starting substrate derived from its availability and the ease of sampling.

## 2. Materials and Methods

### 2.1. Patients 

Between November 2019 and January 2021, all patients suffering from acute uncomplicated diarrhea and evaluated at the Veterinary Teaching Hospital, University of Camerino, were considered to be included in the study. All samplings, the results of which are reported herein, were performed in the interest of the patient; no samplings were performed for the exclusive purposes of the study. In all cases, owners signed an informed consent (Institutional Animal Welfare Body, University of Camerino: prot. no. 1D580.26/A).

### 2.2. Inclusion/Exclusion Criteria

Considering that patients with spontaneous disease were included, the aim of inclusion/exclusion criteria was to reduce as much as possible at least those factors that could have influenced their intestinal environment before the present episode. More precisely, patients that had been treated with antimicrobials and/or probiotics and/or anti-inflammatory drugs in the previous 3 months were excluded from the study. Dogs referring episodes of acute diarrhea in the previous 3 months were also excluded. At fecal coprological examination, including the search for *Giardia*, all patients enrolled tested negative. The need for antimicrobial treatment for the present episode (e.g., cases showing signs of sepsis and/or systemic inflammatory response syndrome), considered as a possible further “confounding factor”, was also considered an exclusion criterion.

### 2.3. Sampling and Patients’ Management

Naturally voided fecal samples were collected immediately after evacuation and then stored at −20 °C until preparation for electrophoresis. Three different time points were considered for fecal collection: T0—day of the first visit/inclusion; T1—after 48 h; T2—14 days after the first presentation. At the same time points, the fecal scores (FS) were also recorded (in brief: grade 1—dry; 2—normal; 3—soft conformed; 4—soft not conformed; 5—watery diarrhea) [16]. All patients variably underwent clinical, diagnostic imaging and laboratory (e.g., blood count and blood chemistry, and fecal parvovirus/coronavirus) evaluations that were deemed necessary to diagnose and manage the single conditions. As therapeutic management, all subjects were variably treated with a standard/symptomatic approach (e.g., dietary variations, fluid therapy, probiotics, antiemetics, and antacids) [3,17], according to the individual clinical condition and to the presence of any comorbidities (e.g., dehydration, vomiting). The probiotic mixture administered was represented in all cases by SivoMixx^TM^ (Ormendes SA, Jouxtens-Mézery, Switzerland), containing eight bacterial strains: *Streptococcus thermophilus* DSM 32245/CNCM I-5570, *Lactobacillus brevis* DSM 27961/CNCM I-5566, *Bifidobacterium lactis* DSM 32246/CNCM I-5571, *Bifidobacterium lactis* DSM 32247/CNCM I-5572, *Lactobacillus plantarum* DSM 32244/CNCM I-5569, *Lactobacillus paracasei* DSM 32243/CNCM I-5568, *Lactobacillus acidophilus* DSM 32241/CNCM I-5567, and *Lactobacillus helveticus* DSM 32242/CNCM I-5573. The amount administered was 100 billion bacteria once per day for dogs from 5 to 10 kg/bw and 200 billion bacteria once per day for dogs ≥10 kg/bw, for at least 14 days.

### 2.4. 2-DE and Mass Spectrometry Analysis

Two grams of frozen feces from each patient was weighed and pooled together. The experimental design of the present work was based on the complete sample pooling strategy as described in previous works [18,19]. The fecal pool was subsequently diluted by adding three volumes (1:3) of phosphate-buffered saline (PBS) containing a 1:100 diluted protease inhibitor cocktail (Sigma-Aldrich, Saint Louis, MO, USA) and incubated with agitation on an ice bath for 30 min. After centrifuging the sample at 10,000 rpm for 20 min, the supernatant was recovered and filtered three times with a filter paper, then with a 0.45 µm filter, and finally with a 0.22 µm filter (Whatman, Maidstone, UK), to remove contaminants and impurities. Ninety percent ammonium sulphate (NH_4_)_2_SO_2_ (Sigma-Aldrich, Saint Louis, MO, USA) was slowly added to the filtered sample, keeping the sample on ice and stirring it for 30 min. After the incubation time, the sample was centrifuged at 13,000 rpm (microfuge) for 30 min. The supernatant was removed, the precipitate was resuspended in PBS, and the protein content was determined by the Bradford method [20]. After protein determination, 1.0 mg of total protein was treated with the 2DClean-Up Kit (GE-Healthcare Life Sciences, Uppsala, Sweden) to remove contamination before the electrophoretic run. The pellet obtained after 2DClean-Up Kit treatment was resuspended in 350 µL of the rehydration buffer containing 8 M urea; 2% (*w*/*v*) 3-[(3-Cholamidopropyl)-dimethylammonio]-1-propanesulfonate (CHAPS); 65 mM dithiothreitol (DTT), 0.001% (*w*/*v*) bromophenol blue, and 0.5% (*v*/*v*) IPG buffer (pH 3–10). 2-DE was performed as previously described [9]. Isoelectrofocusing (1st dimension) was carried out using a pre-cast immobilized pH gradient gel strip (Immobiline DryStrip, IPGstrip, length 18 cm, pH range of 3–10) through an IPGphor isoelectric focusing cell, GE-Healthcare); the 2nd dimension was 13% SDS-PAGE, carried out by a Protean II apparatus (Bio-Rad, Hercules, CA, USA). The gels (180 × 200 × 1.5 mm) were run at 30 mA per gel for 6–7 h. After SDS-PAGE, the gel was stained (0.1% Coomassie Brilliant Blue R250, 50% CH_3_OH; 10% CH_3_COOH), destained (50% CH_3_OH; 10% CH_3_COOH), scanned at 600 dpi, and then analyzed by the PDQuest software (Version 7.1.1; Bio-Rad Laboratories, Segrate (MI), Italy) to calculate the isoelectric point (pI), the molecular mass, and the normalized quantity of each spot in the gel. The pIs were determined using a linear 3–10 distribution, and molecular mass determinations were based on the marker Prestained Protein SHARPMASS VII (6.5–270 kDa; Euroclone s.p.a., Pero (MI), Italy). After gel destaining, the spots were excised from the gel, and the proteins were extracted after being trypsinized, following the protocol described by Shevchenko et al. [21]. The resulting tryptic peptides were resuspended in 100 µL of 0.1% (*v*/*v*) trifluoroacetic acid and analyzed with a UHPLC-HRMS (Agilent Technologies 1290 Infinity II, Q-TOF G6545B, Santa Clara, CA, USA). The column was a reversed phase Luna Omega 1.6 µm Polar 100 Å, 100 × 2.1 mm (Phenomenex, Torrance, CA, USA) kept at 45 °C. The mobile phase was composed of water (A) and acetonitrile (B), both with 0.1% of formic acid. The separation was performed at 0.2 mL/min with the following gradient: 0 min, 2% B; 2.9 min, 2% B; 5 min, 5% B; 31 min, 14% B; 63 min, 22.5% B; 83 min, 36% B; 84 min, 36% B; 86 min, 81% B; 92 min, 81% B. The ion source parameters were: gas temperature 250 °C, drying gas 12 L/min, nebulizer 35 psi, sheath gas temperature 275 °C, sheath gas flow 12 L/min, VCap 3500 V, nozzle voltage 1000 V and fragmentor 100 V. The spectra were obtained in positive ionization mode in auto MS/MS with variable collision energy (slope 3.6, offset −4.8); acquisition range 300–2200 m/z. The obtained MS spectra were extracted and analyzed by the MASCOT software (http://www.matrixscience.com/, accessed on 10 December 2022) with the following search parameters: database, Swiss-Prot; Taxonomy, *Mammalia*; Enzyme, trypsin; peptide tolerance, 1.2 Da; MS/MS tolerance, 0.6 Da; and the allowance of one missed cleavage.

### 2.5. Statistical Analysis

Two-dimensional electrophoresis analyses were performed at least in triplicate. Data were analyzed by using GraphPad Prism^®^ 6.01 software. One-way ANOVA with Tukey correction for multiple comparisons was employed when three or more groups were compared. Significant differences between means were indicated when *p* < 0.05.

## 3. Results

Considering the strict inclusion/exclusion criteria envisaged by the present study, it was possible to include 9 patients suffering from spontaneous acute uncomplicated diarrhea; data related to breed, age and sex are reported in Table 1. Due to the dropout of patient n. 3, we had the opportunity to collect fecal samples at all three predicted time points in 8 out of 9 dogs; for dog n. 3, we obtained a fecal sample only at T0, and therefore, it was not included in the fecal proteomic evaluation. In all cases, the presumptive etiologic diagnosis was of toxinfection likely associated with food poisoning ad/or scavenging activity. The therapeutic approach consisted of the administration of probiotics in all cases, dietary changes in almost all cases (7/9) (see Table 1), and then, variably, of the administration of antisecretory drug (ranitidine; 4/9), antiemetics (maropitant or metoclopramide; 3/9), fluid therapy (1/9), and complementary feed to support liver function (1/9) (Table 1). Table 1 also reports serum albumin values at the time of inclusion, as they will be later analyzed in the discussion. Considering that, as reported above, no samplings were performed for the exclusive purposes of the study, such values were not available for all included patients at T0, and in no case at T1 or T2.

### Fecal Proteomics

**Figure 1:**
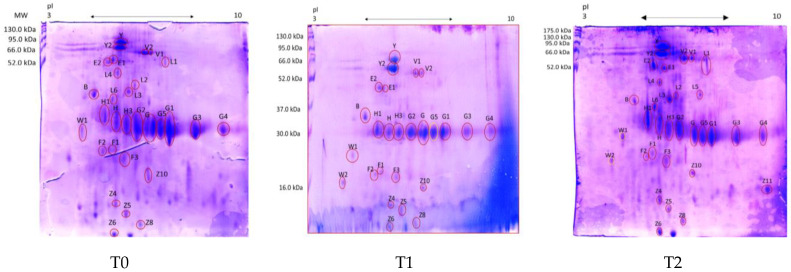
2-DE map of fecal proteins from patients affected by AUD at T0, T1 and T2. The proteins were separated on an immobilized pH 3–10 linear gradient strip and subsequently subjected to 13% SDS-PAGE. Evidenced in red are the spots identified by the mass spectrometry analysis. The protein standards were Prestained Protein SHARPMASS VII (6.5–270 kDa).

**Figure 2:**
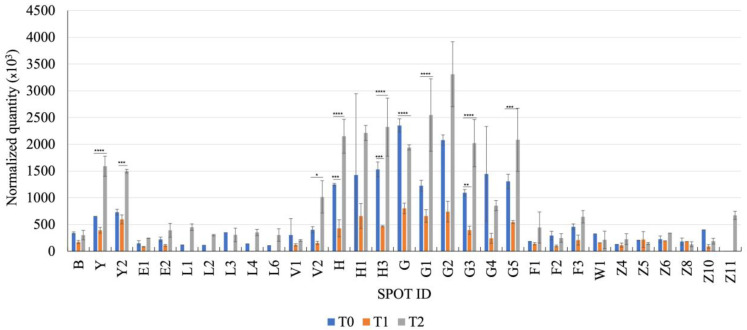
Quantitative analysis of each spot in the feces of dogs affected by acute uncomplicated diarrhea at T0, T1, and T2 determined by the PDquest software. Each spot was identified as indicated in Table 1. Data are shown as mean values ± SE. **** *p* < 0.0001, *** *p* < 0.001, ** *p* < 0.01, * *p* < 0.05.

Figure 1 shows the 2-DE map of fecal proteins extracted from patients affected by AUD at T0 compared to patients at T1 and T2, whereas Table 2 shows the proteins identified by mass spectrometry analysis together with their molecular mass (Mr, kDa) and isoelectric point (pI). Figure 2 shows the normalized quantity calculated for each spot by the PDQuest software analysis. The proteomic map of AUD dogs’ feces at T0 was also compared to the fecal proteome of healthy dogs previously reported in the literature and prepared with the same methodology [9] (see Table 2 and discussion).

## 4. Discussion

The present study reports for the first time (to the authors’ knowledge) the evaluation of the fecal proteome in dogs suffering from acute diarrhea, and it describes for the first time such investigation in the same patients, at different time points, in such a way as to evaluate the evolution of the same condition. 

As reported above, the fecal proteomic evaluation was performed on 8 (out of 9) dogs included in the study. Analyzing results for proteins that significantly differed in content at two or more of the three time points, we first found spots Y and Y2, corresponding to serum albumin isoforms, that were present in all of the samples under analysis. What looked very interesting about this protein was that two days after the onset of the clinical condition (T1), it decreased with respect to T0 (although not significantly) but then significantly increased at T2 (T0 vs. T2, *p* < 0.0001 and *p* < 0.001, respectively, for Y and Y2). Indeed, high content of fecal albumins was reasonably expected at T0, due to the acute mucosal damage and plasma protein loss, as well as a likely progressive decrease at T1 and T2, in parallel with the mucosal healing, which instead only occurred at T1. It is very difficult to comment on such data, as the risk of being excessively speculative is extremely high, and it necessarily needs further confirmation. However, it should be taken into consideration that the two day interval between T0 and T1, adopted in the present study, could be insufficient to reveal evident differences in the GI environment, also due to an impaired GI transit time; therefore, hereafter we discuss mainly the differences between T0 and T2. Nevertheless, for albumin, we should consider what is known about the rapid, almost complete, renewal of the functional villus epithelium by the stem cells of the crypts of Lieberkühn every 2 to 6 days [22] in most adult mammals, and that mathematical modeling suggests that an estimated 1400 mature enterocytes are shed from a single villus tip in each 24 h period [23]. Therefore, it could be reasonable to assume that this physiological rate of cell (and albumin) loss, due to mucosal renewal, may increase very severely during AUD (in our case, T0), due to a pathologic increase in desquamation along the entire surface of the villi. Subsequently, during the repair phase (in our case, T1), a sharp decrease in cell desquamation (and albumin loss) could be normal, since the new cells generated from the crypts of Lieberkühn must re-epithelialize the mucosa covering the villi, reducing the number of mature cells that normally desquamate [24]. Finally, once the complete repair and re-epithelialization of the villus take place, the cellular desquamation will return to a normal level for a substantial recovery of the physiological cellular turnover of the enterocytes, which could even be accelerated by the healing process (in our case, T2) [22]. It was then also noticeable that the amount (normalized quantity) of the above two spots was found to be considerably higher in the feces of AUD dogs at T0 compared to the fecal samples of healthy dogs [9], respectively 652 ± 2 × 10^3^ and 729 ± 54 × 10^3^ versus 115 ± 47 × 10^3^ and 79 ± 13 × 10^3^, as reasonably expected in this case due to the onset of the mucosal damage in the diseased patients. Proceeding further, two isoforms of alkaline phosphatase (AP) (spots V1 and V2) were consistently observed in all samples (T0, T1 and T2). In the case of spot V2, it decreased at T1 but then significantly increased at T2 (*p* < 0.05). The intestinal AP is an enzyme index of enterocyte maturation and has an anti-inflammatory activity through the degradation of bacterial lipopolysaccharide (LPS). It has been noticed to be decreased in patients with chronic enteropathies likely due to dysbiosis, which in turn reduces the enzyme’s degradative activity [25,26]. It is interesting to underline that this protein was also found with a proteomic approach in the feces of healthy dogs and dogs suffering from intestinal lymphangiectasia, food-responsive diarrhea and chronic inflammatory enteropathies, and that in all of these cases, the amount in diseased patients was lower than in healthy ones [9,10,12]. In the cases reported herein, it is reasonable to assume that the increase in the enzyme at T2 could derive from the regenerative process and the maturation of the enterocytes. Spot H was attributed to Chymotrypsin-C-like. Chymotrypsin C normally degrades trypsinogen limiting; therefore, its intrapancreatic activation and protection against pancreatitis [27,28]. A recent fecal proteomic study performed in human medicine suggested for this protein a role as a biomarker in patients suffering from IBD [29]. This protein was also previously found in the feces of healthy dogs and dogs suffering from intestinal lymphangiectasia and chronic enteropathies [9,10,12]. Similarly to the proteins previously described, also in this case it decreased (significantly) at T1 and increased (significantly) at T2, but it is difficult to unravel this trend. Finally, the remaining proteins differing at two or more of the three time points were all immunoglobulins (H3: Immunoglobulin kappa light chain; G and G1: Immunoglobulin λ-1 light chain; G3: Immunoglobulin λ-light chain VLJ region; G5: Immunoglobulin kappa chain V region). As before, in all cases, the content of such proteins decreased from T0 to T1 and then significantly increased at T2 (in all cases except spot G). Many studies have shown that enterocytes have the ability to concentrate large quantities of immunoglobulins in their cytoplasm [30] and that they could be released through the basolateral and apical routes at the level of the brush border of the enterocytes [31]. It is, therefore, normal that in the acute phase of diarrhea (T0), we found a high quantity of these proteins in the feces, as, like albumin, they were reasonably released by damaged enterocytes that detached from the surface of the villi. It is also conceivable that this condition slowed down successively during the reparative phase of the mucosa (reduction at T1), returning to growth after 14 days (T2) when the production of immunoglobulin associated with the activation of the body’s immune response had been added to the normal, restored, cellular turnover (with the “physiological” release of the intracellular IgG) [24]. It is honestly difficult to interpret why the immunoglobulin of spot G was higher at T0 if compared to both T1 and T2, although it is, however, a single finding. It should then also be noticed that all of these proteins, except the Immunoglobulin kappa chain V region (spot G5), were previously found in healthy dogs [9], demonstrating that their excretion in feces is related to normal enterocyte turnover. Among proteins not differing significantly at any time point, spots L2, L3, L4, and L6 emerged in the AUD samples but not in a previous evaluation in healthy dogs performed with the same methodology [9]; they corresponded to albumin and presumably were isoforms of this protein, as reported in the literature [32]. Additionally, spots Z (fatty acid-binding protein) and Z10 (superoxide dismutase [Cu-Zn]) were found in healthy dogs and in dogs suffering from chronic enteropathies, with Z10 being of particular interest since it is an antioxidant enzyme that acts during oxidative stress [12,33].

Proteomic results regarding albumin were even more interesting if compared to the serum albumin concentrations in the study group. For our patients, we had the albumin values at T0 available for 6 out of 9 dogs, and in all cases, except for patient no. 1 in which the value was 0.05 g/dL, below the reference range (2.6 g/dL), such values were within the normal range, leading to the simultaneous presence of albumin in the stool and to the absence of hypoalbuminemia. This interesting datum agrees with a previous study in a different species, using vein inoculation of radioactively labeled albumin, showing that the albumin found in the feces does not derive from plasma unless the enteropathy is specifically a (chronic) protein-losing enteropathy [34]. Nevertheless, the increase in fecal albumin in AUD patients is also aligned with the recent study of Leipig-Rudolph et al., who investigated intestinal lesions in mucosal biopsies from dogs with acute hemorrhagic diarrhea syndrome [6], highlighting lesions of different severity (from mild to severe) in almost all patients, findings that agree with the possibility of fecal plasmatic albumin loss during acute diarrhea, as also previously reported, although always in hemorrhagic cases, by Heilmann et al. [5]. On the other hand, always accordingly with our findings (i.e., absence of hypoalbuminemia), it should be noticed that hypoalbuminemia (likely due to significant intestinal loss of plasma protein) is usually found only in less than 10% of dogs with AHDS [35,36]. Due to the nature of the study, at T1 or T2 we did not have the opportunity to analyze blood samples, and therefore, to measure serum albumin, in any of the patients.

Finally, with regard to the clinical evolution of the FS, all dogs included in the study presented a complete resolution with normal (6 cases), if not dry (1 case), stools 14 days after the onset of clinical signs (T2); the only patient (no. 2) that presented a grading between normal and soft conformed (score 2.5) was, however, considered clinically resolved. On the other hand, 2 days after the clinical presentation (T1), although the mean value of the fecal scores of all of the patients included in the study was improved, decreasing from 3.8 at T0 to 3.2 at T1, only 2 out of 8 patients presented normal stools, while the remaining 6 still had fecal scores ≥ 3. Nevertheless, the FS was improved in 5 dogs (with a total reduction in the scores equal to 6.5, mean of 1.3), remained unchanged in one, and worsened in two (of 0.5 each). If we compare these data with the previous literature on different kinds of canine acute diarrheas, it is interesting to notice that improvements/significant clinical recovery were achieved in 1.3 to 6.6 days [36,37,38,39,40]. Considering the above, although different monitoring time points were used in the present study, and a recent review on that topic referring that in most cases of acute hemorrhagic diarrhea syndrome in dogs the clinical recovery occurs within 24 and 72 h [35], the clinical trend reported herein appears to be in line with the previous literature.

The main weaknesses of the present study are represented by: (1) the small number of patients included, due to the strict inclusion/exclusion criteria; (2) the slightly different therapeutic approach administered (including diet), that depended on the severity and on the different clinical signs reported, and in turn was strictly related to the third weakness represented by (3) the fact that patients were all spontaneously affected by AUD whose etiology was not diagnosed, although all of the pathological conditions were reasonably traced back to toxinfection/food poisoning/scavenging activity. The apparently strange finding of the study that most spots decreased at T1 and increased at T2 was not due to artifacts related to the experimental procedure, since some proteins (e.g., Z5, Z8, and Z11 [Hemoglobin subunit beta]) behaved differently at the three time points considered. As previously speculated, it is possible that the two-day interval between two fecal samplings to be used for proteomic evaluations and adopted in the present study (T0 to T1) could be too short to appreciate fine changes in the proteome considering the impaired transit time. However, in a couple of cases (i.e., albumin and immunoglobulins), we had the opportunity to interpret this trend; therefore, this aspect needs to be further explored.

Lastly, beyond the primary objectives of the present study, considering the patients’ management and evolution, they further reaffirm, if ever there was still a need, that the use of antimicrobials in canine acute diarrhea, as well as in chronic cases [41], is not the first option.

## 5. Conclusions

The present study reported for the first time the evaluation of the fecal protein profile in dogs suffering from acute uncomplicated diarrhea and such assessment at different time points, providing insights into what happens inside the intestine during the evolution of these pathological conditions. Four (groups of) proteins—albumin, alkaline phosphatase, chymotrypsin-C-like, and some immunoglobulins—showed significant differences at two or more of the three time points investigated, almost all behaving similarly and decreasing at T1 (2 days after the onset of the disease) and significantly increasing at T2 (14 days after onset), mainly suggesting a reaction of the organism. It needs to be additionally investigated whether a two-day period between two fecal samplings to be used for fecal proteomic evaluations is adequate to highlight significant changes. The results reported herein need to be further confirmed in future studies including a greater number of patients and possibly different techniques.

## Figures and Tables

**Table 1:** Clinical data, fecal score (FS), and serum albumin values (at T0) of the patients included in the study.

N.	Breed	Age (Years)	Sex	Therapy Undertaken	FS ^§^ T0	FS ^§^ T1	FS ^§^ T2	* Serum Albumin (g/dL) [Reference Value 2.60–3.30]
**1**	English Setter	2	M	Diet ** + probiotics	4.5	5	2	*2.55*
**2**	Pinscher	4	M	Diet ** + probiotics + ranitidine + maropitant + complementary feed to support liver function	3.5	4	2.5	*3.56*
**3**	Cocker Spaniel	3	M	Probiotics + fluids + ranitidine + maropitant	3	-	-	*2.67*
**4**	Labrador Retriever	2	F	Probiotics + ranitidine + metoclopramide	4	3	2	*3.7*
**5**	Labrador Retriever	7	F	Diet ** + fluids + probiotics + ranitidine	3	3	2	*2.7*
**6**	Australian Shepherd	5	M	Diet ** + probiotics	5	4.5	2	*-*
**7**	Border Collie	9	F	Diet ** + probiotics	4	3	2	*-*
**8**	Mixed breed	10	M	Diet ** + probiotics	4	1	1	*-*
**9**	Mestizo Pinscher	2	F	Diet ** + probiotics	3	2	2	*2.86*
**Mean values**					**3.8**	**3.2**	**1.9**	***3.00***

^§^ FS = Fecal score [16]; * Values at T0, for those patients in which it was available; ** Diet variably means: homemade diet (potatoes plus boiled turkey breast), commercial gastrointestinal or single protein diet.

**Table 2:** Identification of fecal proteins in dogs suffering from AUD by LC-MS/MS followed by MASCOT and SONAR software analysis (www.matrixscience.com [accessed on 15 September 2022]) at T0.

Spot ID ^a^ Healthy	Spot ID ^b^ AUD	Protein ^c^	Score ^d^	Mr (kDa)/pI ^e^	Mr (kDa)/pI ^f^	Sequence
n.d.	B	Albumin [*Canis lupus familiaris*]	39	68.5/5.52	35.7/4.8	DFAEISK
present	Y	**Serum albumin isoform X1 [*Canis lupus familiaris*]**	56	68.6/5.51	72/5.8	LVAAAQAALV
present	Y2	**Serum albumin isoform X1 [*Canis lupus familiaris*]**	41	68.6/5.51	63/5.8	ADFAEISK
n.d.	E1	Albumin [*Canis lupus familiaris*]	216	68.5/5.52	53.3/5.3	DFAEISKVVTDLTK
n.d.	E2	Albumin [*Canis lupus familiaris*]	104	68.5/5.52	50.7/5.2	KLGEYGFQNALLVR
n.d.	L1	Intestinal-type alkaline phosphatase [*Mus musculus*]	103	60.2/6.24	52.3/7.2	NLIIFLGDGMGVPT
n.d.	L2	Albumin [*Canis lupus familiaris*]	131	68.5/5.52	36.1/6.1	LVAAAQAALV
n.d.	L3	Albumin [*Canis lupus familiaris*]	332	68.5/5.52	33.8/5.8	LVAAAQAALV
n.d.	L4	Albumin [*Canis lupus familiaris*]	131	68.5/5.52	43.8/5.5	LVAAAQAALV
n.d.	L6	Albumin [*Canis lupus familiaris*]	100	68.5/5.52	29.9/5.3	DFAEISKVVT
present	V1	Alkaline phosphatase [*Canis lupus familiaris*]	125	68.6/6,47	59/6.6	ANYQTIGVSAAAR
present	V2	**Alkaline phosphatase [*Canis lupus familiaris*]**	117	48.3/6.15	58/6.6	ANYQTIGVSAAAR
present	H	**Chymotrypsin-C-like [*Canis lupus dingo*]**	49	29.1/5.33	29/5.6	LAEPVQLSDTIK
present	H1	Elastase-3B, Proteinase E [*Canis lupus familiaris*]	40	28.8/5.27	29/5.2	VSAFNDWIEEVMSS
present	H3	**Immunoglobulin kappa light chain [*Felis catus*]**	41	26.7/6.10	29/6.3	FSGSGSGTDFTLR
present	G	**Immunoglobulin λ-1 light chain [*Canis lupus familiaris*]**	34	25.2/6.88	29/7.1	KGTHVTVLGQPK
present	G1	**Immunoglobulin λ-1 light chain [*Felis catus*]**	39	27.8/8.17	29/7.6	QSNNKYAASSYL
present	G2	Immunoglobulin λ-light chain VLJ region [*Homo sapiens*]	42	29.0/8.14	29/6.6	EFGGGTKLTVLGQP
present	G3	**Immunoglobulin λ-light chain VLJ region [*Homo sapiens*]**	30	29.0/8.14	27/8.4	EFGGGTKLTVLGQP
present	G4	Immunoglobulin λ-light chain VLJ region [*Homo sapiens*]	40	29.0/8.14	27.7/8.9	QSNNKYAASSYL
n.d.	G5	**Immunoglobulin kappa chain V region [*Canis lupus familiaris*]**	84	12.0/6.4	25/7	FSGSGSGTDFTLR
present	F1	Nuclear pore membrane glycoprotein 210 [*Canis lupus familiaris*]	29	192.4/6.30	19.6 ± 1.5/5.8 ± 0.14	TALLVTASISGSHAPR
present	F2	Cytosol aminopeptidase [*Canis lupus familiaris*]	29	56.2/8.03	21.0 ± 1.3/5.7 ± 0.07	EILNISGPPLK
n.d.	F3	Immunoglobulin lambda variable 4–60 [*Homo sapiens*]	69	12.9/5.8	16.3 ± 1.6/6 ± 0.1	FSGSSSGADR
n.d.	W1	Immunoglobulin J chain [*Homo sapiens*]	43	18.1/5.12	21 ± 0.07/4.1 ± 0.05	IIVPLNNR
n.d.	Z4	Albumin [*Canis lupus familiaris*]	152	68.5/5.52	8.8 ± 0.5/4.1 ± 0.03	LVAAAQAALV
n.d.	Z5	Fatty acid-binding protein, intestinal [*Homo sapiens*]	69	15.2/6.6	8.3 ± 3.4/5.9 ± 0.07	LTITQEGNK
n.d.	Z6	Albumin [*Canis lupus familiaris*]	76	68.5/5.52	7.1 ± 3.1/5.5 ± 0.2	EAYKSEIAHRYNDLGE
n.d.	Z8	Albumin [*Canis lupus familiaris*]	80	68.5/5.52	8.1 ± 0.1/6 ± 0.1	EAYKSEIAHRYNDLGE
n.d.	Z10	Superoxide dismutase [Cu-Zn] [*Canis lupus familiaris*]	233	15.9/5.7	8.8 ± 0.5/6.2 ± 0.1	EKRDDLGKGDNEEST

^a^ From data previously published for healthy dogs, obtained with the same methodology [9]; ^b^ Spots assigned as indicated in Figure 1; ^c^ MASCOT results (Swiss-Prot and NCBInr databases); ^d^ MASCOT Score; ^e^ Swiss-Prot and NCBInr databases; ^f^ Experimental values calculated from the 2-DE maps by the PDQuest software (mean ± standard deviation); **Note: highlighted in bold** are proteins that significantly differed in content at two or more of the three time points (T0, T1, and T2); AUD: acute uncomplicated diarrhea; n.d.: not detected.

## Data Availability

All relevant data are contained within the article.
